# Deposition of collagen type I onto skeletal endothelium reveals a new role for blood vessels in regulating bone morphology

**DOI:** 10.1242/dev.139253

**Published:** 2016-11-01

**Authors:** Adi Ben Shoham, Chagai Rot, Tomer Stern, Sharon Krief, Anat Akiva, Tali Dadosh, Helena Sabany, Yinhui Lu, Karl E. Kadler, Elazar Zelzer

**Affiliations:** 1 Department of Molecular Genetics, Weizmann Institute of Science, Rehovot 76100, Israel; 2 Department of Structural Biology, Weizmann Institute of Science, Rehovot 76100, Israel; 3 Irving and Cherna Moskowitz Center for Nano and Bio-Nano Imaging, Department of Chemical Research Support, Weizmann Institute of Science, Rehovot 76100, Israel; 4 Wellcome Trust Centre for Cell-Matrix Research, Faculty of Biology, Medicine and Health, University of Manchester, Manchester M13 9PT, UK

**Keywords:** Collagen type I, Endothelial cell, Vascular patterning, Basement membrane, Endochondral bone formation, Morphogenesis, Osteoid, Mineralization, Mouse, *Vegfa*

## Abstract

Recently, blood vessels have been implicated in the morphogenesis of various organs. The vasculature is also known to be essential for endochondral bone development, yet the underlying mechanism has remained elusive. We show that a unique composition of blood vessels facilitates the role of the endothelium in bone mineralization and morphogenesis. Immunostaining and electron microscopy showed that the endothelium in developing bones lacks basement membrane, which normally isolates the blood vessel from its surroundings. Further analysis revealed the presence of collagen type I on the endothelial wall of these vessels. Because collagen type I is the main component of the osteoid, we hypothesized that the bone vasculature guides the formation of the collagenous template and consequently of the mature bone. Indeed, some of the bone vessels were found to undergo mineralization. Moreover, the vascular pattern at each embryonic stage prefigured the mineral distribution pattern observed one day later. Finally, perturbation of vascular patterning by overexpressing *Vegf* in osteoblasts resulted in abnormal bone morphology, supporting a role for blood vessels in bone morphogenesis. These data reveal the unique composition of the endothelium in developing bones and indicate that vascular patterning plays a role in determining bone shape by forming a template for deposition of bone matrix.

## Abstract (precis)

**Highlighted article:** Collagen I is deposited by osteoblasts onto endothelial cells within bone and serves as a template for mineralisation, with ossification thus spatially and temporally following vascular patterning.

## INTRODUCTION

In vertebrates, long bones develop by a process known as endochondral ossification, where initially chondrocytes form avascularized cartilaginous templates of the future bones. As development proceeds, chondrocytes in the center of the cartilage differentiate to hypertrophy and secrete angiogenic factors, which induce the invasion of blood vessels from the perichondrium into the hypertrophic zone (Zelzer et al., 2004). Osteoclasts, osteoblast and hematopoietic progenitors carried by the invading vessels form the primary ossification center. Concurrently, the first capillary plexus continues to sprout longitudinally toward the ends of the bone. This leads to expansion of the marrow cavity from the center and the formation of the epiphyseal growth plates at both ends, where the cartilage is gradually replaced by ossified bone and bone marrow (Berendsen and Olsen, 2015; Kozhemyakina et al., 2015; Kronenberg, 2003; Lefebvre and Bhattaram, 2010; Provot and Schipani, 2005).

During ossification, osteoblasts form osteoids by depositing bone matrix, predominantly collagen type I encoded by *Col1a1* and *Col1a2* genes. Subsequently, the osteoid is impregnated with hydroxyapatite to form mineralized bone tissue (Boonrungsiman et al., 2012; Landis, 1999; Scherft, 1978). By serving as a template for mineral deposition, osteoids play an important role in bone morphogenesis. However, little is known about the mechanism that regulates the shape and size of this important structure.

Blood vessel invasion into the zone of hypertrophic chondrocytes is essential for bone ossification. Both surgical and genetic inhibition of vessel sprouting in the growing bone result in blocked cartilage erosion, an enlarged hypertrophic zone, and reduced ossification and longitudinal growth (Gerber et al., 1999; Trueta and Amato, 1960; Trueta and Buhr, 1963; Trueta and Trias, 1961; Zelzer et al., 2002). Several genes have been implicated in regulating vessel invasion and continuous sprouting during bone growth (reviewed by Maes, 2013 and Zelzer and Olsen, 2005). Vascular endothelial growth factor (VEGF), which is expressed by hypertrophic chondrocytes and induces angiogenesis, was shown to be a central regulator of bone angiogenesis. Blocking the expression of *Vegf* (*Vegfa* – Mouse Genome Informatics) leads to reduced bone angiogenesis and ossification (Gerber et al., 1999; Maes et al., 2010b; Zelzer et al., 2001, 2004, 2002). By contrast, skeletal overexpression of *Vegf* results in increased bone mass (Maes et al., 2010a). Interestingly, although these works demonstrate the importance of blood vessels for bone formation, the mechanism that mediates their contribution remains elusive.

The vascular system serves not only as a transport network that carries nutrients and oxygen to the cells and removes metabolic waste, but also as an active secretor of a variety of signaling molecules that regulate growth, differentiation, patterning, homeostasis and morphogenesis of developing tissues (Cleaver and Dor, 2012; DeLisser et al., 2006; Jakkula et al., 2000; Lazarus et al., 2011). A recent study shows that during postnatal bone growth, endothelial cells (ECs) secrete noggin, an antagonist of bone morphogenetic protein (BMP), under the regulation of Notch signaling. This angiocrine signaling has been implicated in regulation of bone ossification and growth, suggesting that bone ECs may actively regulate bone formation (Ramasamy et al., 2014).

Here, we identify a new role for the bone vasculature as a guiding template for mineral deposition. We show that in developing bones of mouse embryos, blood vessels uniquely lack basement membrane. These vessels are instead covered by collagen type I and gradually undergo mineralization. High correlation between vascular and bone patterning during development, and changes in the pattern of bone ossification following genetic interference with vascular patterning strongly suggest a central role for the vasculature in bone morphogenesis.

## RESULTS

### Collagen type I coats bone vasculature

**Fig. 1.**
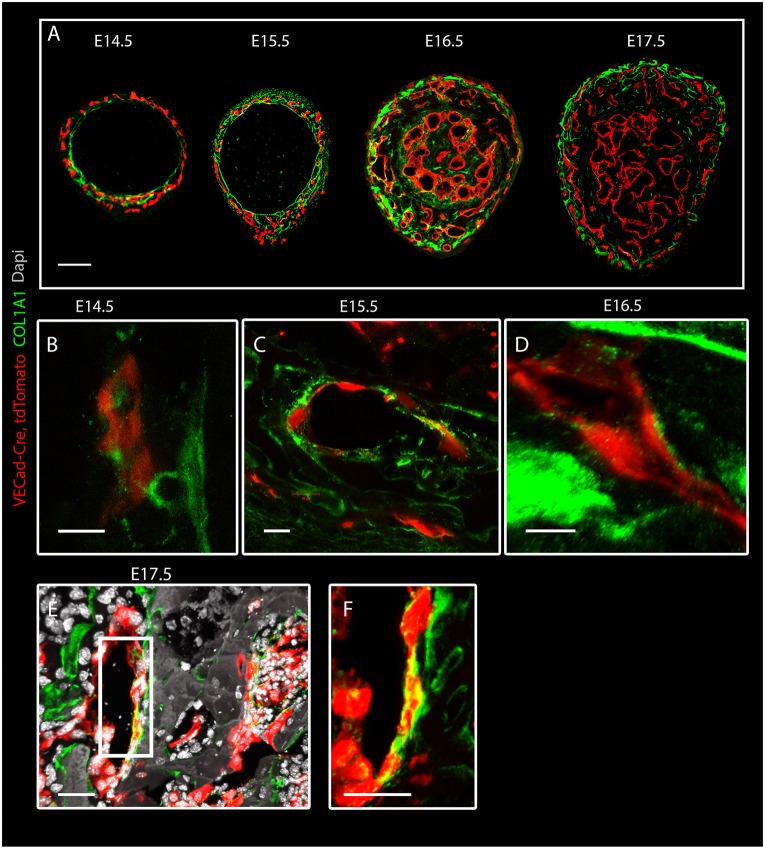
**Collagen type I coats bone vasculature.** Confocal images of humeral cross-sections from *VECad-Cre, tdTomato* (red) mouse embryo immunostained for collagen I (green) at E14.5-E17.5. (A) An overview of vascular and collagen I distribution at different embryonic stages. (B-E) A high concentration of collagen I on ECs is seen at E14.5 (B), E15.5 (C), E16.5 (D) and E17.5 (E). (F) Magnification of the boxed area in E. Scale bars: 100 µm in A; 10 µm in B-D; 30 µm in E,F.

Numerous studies have shown the importance of the coupling of angiogenesis and osteogenesis during bone development (Sivaraj and Adams, 2016). However, the exact role of blood vessels in bone development has remained unknown. Collagen type I is the main fiber type composing the osteoid and serves as a template for mineral deposition. To better understand the association between bone formation and angiogenesis, we studied the correlation between COL1A1 expression and vascular development. To achieve this, the vasculature was visualized by crossing VE-Cadherin-Cre (*VECad-Cre*) mice with *tdTomato* reporter mice; then, immunostaining for COL1A1 was performed at different embryonic stages (E14.5-E17.5) (Fig. 1A). As expected, COL1A1 was observed in the forming osteoid, where bone tissue was forming. Collagen deposition initiated with the formation of the bone collar, a cylindrical layer of collagen around the middle section of the cartilage core. The bone collar then expanded radially through the formation of strut-ring layers, as the outer layers contained newly deposited collagen I.

**Fig. 2.**
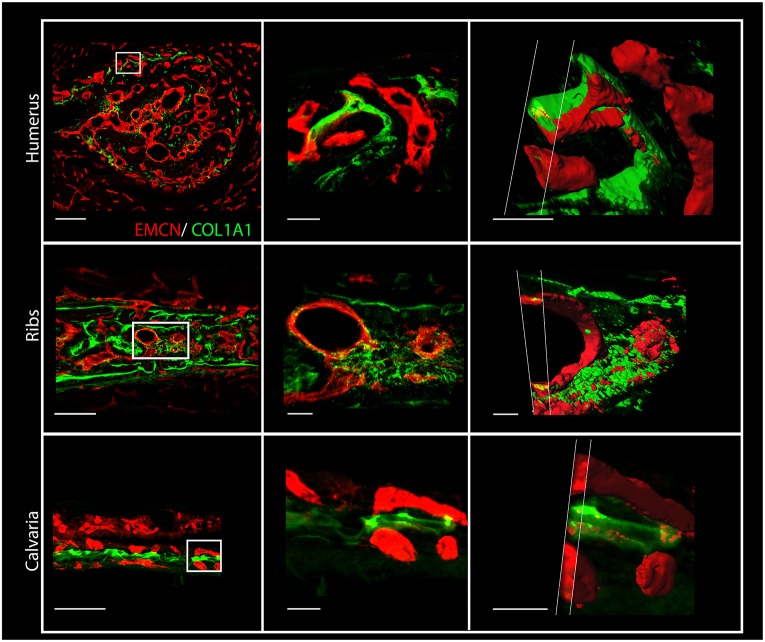
**Collagen type I is deposited on endothelial cells in different bones.** Cross-sections of humeri, ribs and calvaria from an E15.5 mouse embryo immunostained for EMCN (red) and collagen I (green). Middle and right columns show higher magnifications of the boxed areas in the left column. Right column: 3D *z*-stack images of the blood vessels shown in the middle column were cut at different angles to highlight the localization of collagen I on and in ECs. Scale bars: 100 µm, left column; 20 µm, middle; 15 µm, right.

**Fig. 3.**
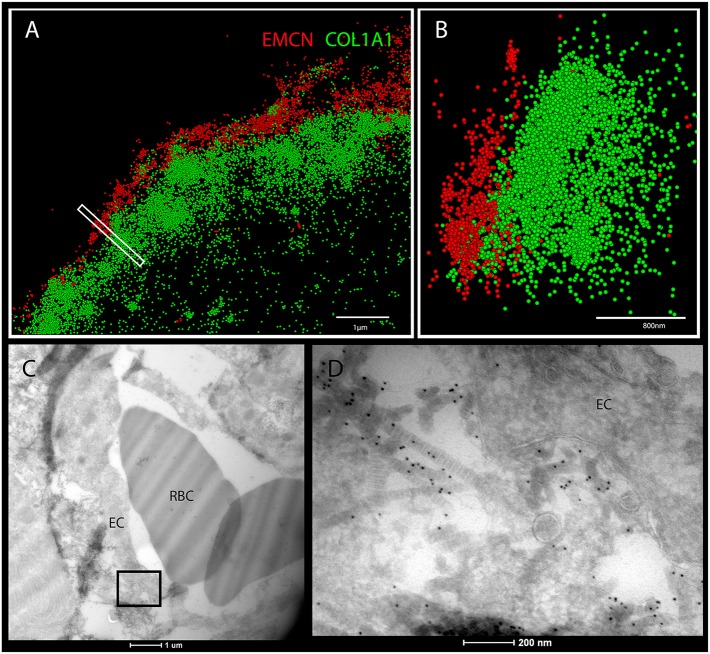
**Super-resolution microscopy and transmission electron microscopy show collagen I on ECs in developing bones.** (A,B) STORM images of cross-sections of humeri at E15.5 immunostained for EMCN (red) and collagen I (green). (B) A side view of the boxed area in A. (C,D) Cross-sections of E16.5 mouse humerus observed by TEM. (C) Cross-sections of blood vessels from the bone area. (D) Magnification of the boxed area in C. Collagen type I immunostained with 10 nm gold particles is located on the endothelial cell (EC) surface. RBC, red blood cells. Scale bars: 1 µm in A; 800 nm in B; 1 µm in C; 200 nm in D.

**Fig. 4.**
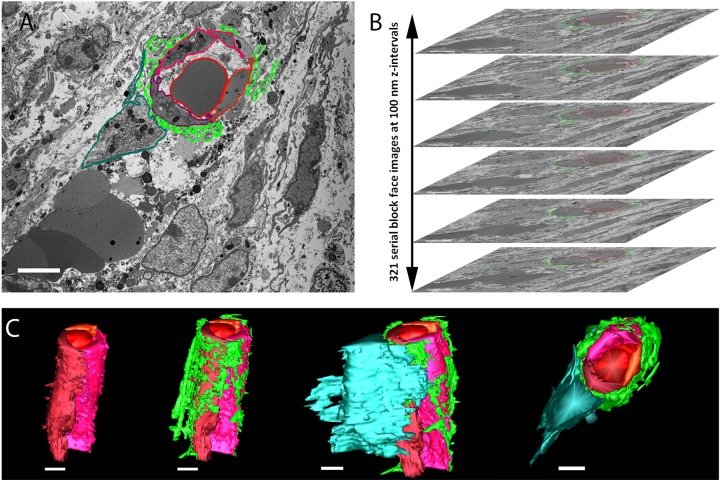
**Serial block-face scanning electron microscopy shows a blood vessel coated with collagen I and associated with an osteoblast.** Cross-sections of humerus from an E16.5 mouse embryo. (A,B) Serial images were obtained at 100 nm intervals along the *z*-axis of the bone. ECs and a red blood cell are demarcated by lines in different shades of red, collagen type I is demarcated in light green and an osteoblast in dark green. (C) Three-dimensional reconstruction using IMOD shows from different angles the same vessel consisting of three ECs and carrying an erythrocyte, which is covered with collagen and is associated with an osteoblast. Scale bars: 5 µm.

Surprisingly, in addition to the osteoid we observed collagen I in close association with ECs, seemingly coating them (Fig. 1B-F). Immunostaining with different sets of antibodies against collagen I and blood vessels further verified this result (Fig. S1). The association of collagen I and ECs was seen in different bones, namely humerus, ribs and calvaria (Fig. 2). As the results were obtained using confocal microscopy, which is limited by the diffraction limit, we used the super-resolution imaging technique stochastic optical reconstruction microscopy (STORM). As seen in Fig. 3A,B, at a resolution of 40 nm tight proximity and even overlap were observed between ECs and collagen I. To verify these observations unambiguously, we performed transmission electron microscopy (TEM) on tissue that was immunogold labeled with anti-collagen I antibody. TEM results revealed small fibrils with the characteristic *D*-periodic (67 nm) banding pattern of collagen fibrils (Kadler et al., 1996), which were stained with gold particles and were found attaching to the EC surface (Fig. 3C,D). Finally, to assess the level of coverage of blood vessels by collagen I, we used serial block-face scanning electron microscopy (SBF-SEM), which produces three-dimensional information at electron microscope resolution by sequential imaging and thin sectioning of embedded tissue (Fig. 4). Analysis of E16.5 humerus revealed extensive coverage of blood vessels with collagen fibrils. In addition, osteoblasts were observed in close association with ECs, whereas collagen fibers were seen filling the gap between them (Fig. 4 and Movie 1). Together, these results clearly demonstrate that, in the developing bone, some blood vessels are extensively coated with collagen type I.

### Osteoblasts but not endothelial cells produce collagen I

Our finding that collagen type I coats ECs led us to speculate on the mechanism of coating. Although bone collagen I is known to be produced by osteoblasts, which may secrete this protein onto ECs, it is possible that bone ECs can also secrete this protein. Alternatively, ECs might transdifferentiate to osteoblasts and then produce collagen I, or it could be that the mural cells that surround blood vessels play a role in the coating of ECs with collagen I.

**Fig. 5.**
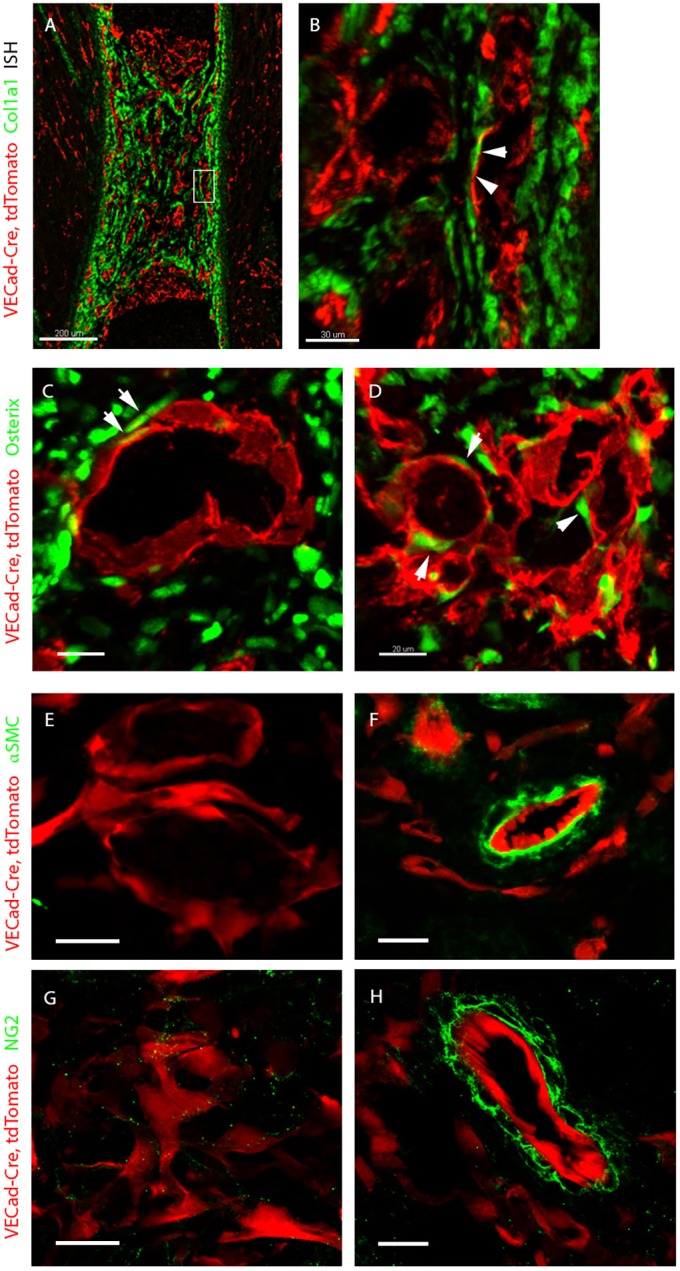
**Collagen I is secreted by osteoblasts onto vessels.** (A,B) *In situ* hybridization for *Col1a1* (green) and *tdTomato* (red) in E16.5 *VECad-Cre, tdTomato* mouse embryo. *Col1a1* is broadly expressed in the perichondrium, but not by ECs. Arrows indicate high proximity of *Col1a1*-positive cells to ECs, with no colocalization. (C,D) Cross-sections of humeri at E15.5 and E16.5 immunostained for the osteoblast marker osterix (green). Arrows indicate the proximity of osteoblasts to ECs. (E-H) Cross-sections of forelimb of a E16.5 *VECad-Cre, tdTomato* mouse embryo at E16.5 immunostained with antibodies against NG2 and α-smooth muscle actin (αSMC) to detect pericytes and smooth muscle cells (green) in the bone vasculature (E,G) when compared with blood vessels outside the bone (F,H). Scale bars: 200 µm in A; 30 µm in B; 20 µm in C-H.

To test the hypothesis that skeletal ECs produce collagen I, we performed *in situ* hybridization for *Col1a1* and *tdTomato* on *VECad-Cre* mice crossed with *tdTomato* reporter mice during the ossification process at E16.5. The results showed a broad expression pattern of *Col1a1* in cells around blood vessels, in the perichondrium and in the primary ossification center, but no expression in ECs (Fig. 5A,B).

To address the possibility of transdifferentiation, we performed lineage-tracing analysis of ECs and their descendants on *VECad-Cre* mice crossed with a *Rosa26-tdTomato* reporter line; osteoblasts were detected by immunostaining against osterix. The results showed very tight spatial proximity between osteoblasts and ECs. Nevertheless, there was no distinct colocalization of ECs and osterix-positive cells, thus negating the notion of endothelial differentiation into osteoblasts (Fig. 5C,D). Finally, in order to test the involvement of mural cells, namely pericytes and smooth muscle cells, in the coating process, we performed immunostaining for NG2 and α-smooth muscle actin in E16.5 *VECad-Cre, Rosa26-tdTomato* mice. The results showed that at this developmental stage, blood vessels in the bone are largely devoid of mural cell covering (Fig. 5E-H). Taken together, these results suggest that collagen I is secreted by osteoblasts onto ECs.

### Blood vessels in developing bones have no basement membrane

**Fig. 6.**
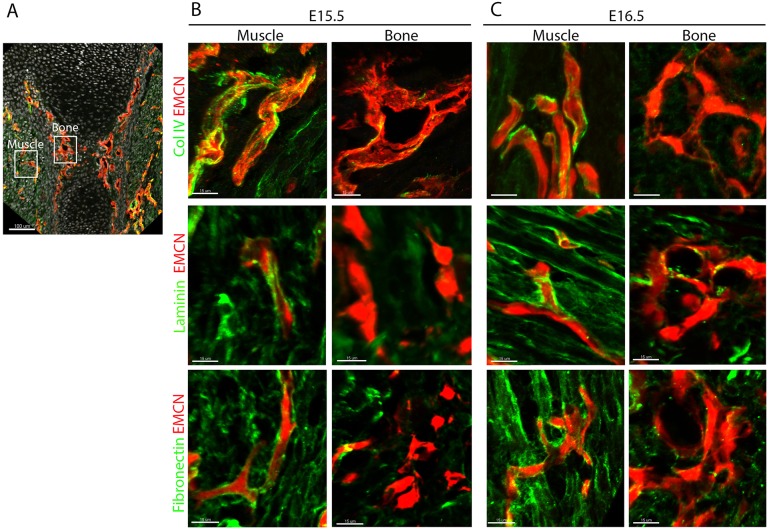
**Blood vessels in the bone lack basement membrane.** (A) Sagittal section of humerus from mouse embryo immunostained for EMCN and for BM markers collagen type IV, laminin and fibronectin. (B,C) Magnifications of the boxed areas in A show muscle and bone at E15.5 and E16.5. Unlike in muscle, blood vessels in bone tissue have no underlying BM. Scale bars: 100 µm in A; 15 µm in B,C.

**Fig. 7.**
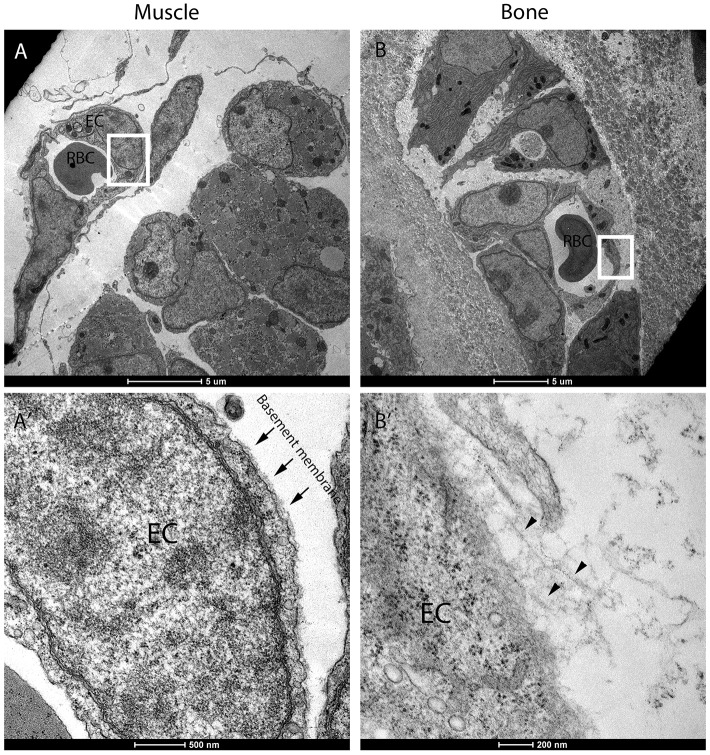
**Transmission electron microscopy shows no basement membrane on ECs in developing bones.** Cross-sections of E16.5 humerus observed by TEM. (A,A′) Blood vessels from the muscle area. Arrows in A′ indicate basement membrane on muscle endothelial cells (ECs), which were absent from bone ECs. (B,B′) Blood vessels from the bone. Arrowheads in B′ indicate fibrillary structure close to the ECs. (A′,B′) Magnification of the boxed areas in A and B, respectively. RBC, red blood cell. Scale bars: 5 µm in A,B; 500 nm in A′; 200 nm in B′.

The basement membrane (BM) is a highly specialized thin layer of extracellular matrix proteins that surrounds blood vessels and thus isolate ECs from their surroundings (Davis and Senger, 2008; Hallmann et al., 2005). As blood vessels are normally surrounded by BM, it was unclear how they could be coated by collagen type I in developing bones. To address this issue, we performed immunostaining for the main BM components collagen IV, laminin and fibronectin on humeri of E15.5 and E16.5 mouse embryos (Fig. 6). As a control, we examined the adjacent muscle tissue, where blood vessels were associated with a characteristic and well-developed BM. By contrast, BM components were completely absent from the endothelium of bone tissue at E15.5 and were observed surrounding only a few vessels at the bone center at E16.5 (Fig. S2), suggesting that the endothelium at the bone circumference and beyond the chondro-osseous junction lacks BM. This observation was further validated by TEM analysis. As seen in Fig. 7, a blood vessel in the muscle was coated with a well-established thin layer of BM and a clear border of the cell membrane was observed. Conversely, in the bone the blood vessels had no BM; instead, we observed a fibril-like structure that connected to the EC surface.

These results demonstrate a unique characteristic of the skeletal endothelium, namely the lack of BM, and provide a mechanistic explanation for the ability of collagen I fibers to coat these vessels.

### The endothelium serves as a template for mineral deposition

**Fig. 8.**
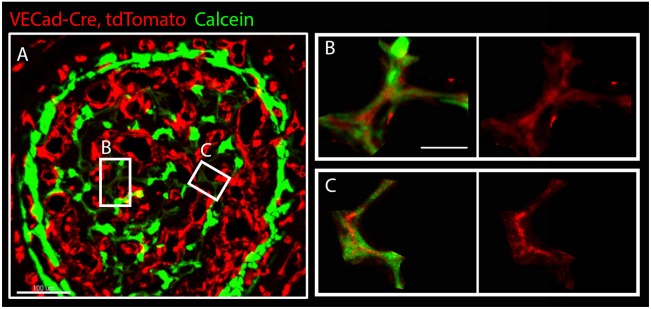
**Low expression of an EC marker in newly mineralized areas.** (A) Cross-section of humerus from *VECad-Cre, tdTomato* (red) mouse embryo at E17.5 injected with calcein (green). (B,C) Magnifications of the boxed areas in A show low expression of *VECad* by ECs in areas of newly deposited mineral, indicated by weak calcein signal. Scale bars: 100 µm in A; 30 µm in B,C.

Collagen I is a key component of the osteoid. Thus, our finding that vessels in developing bones are coated with collagen I led us to hypothesize that collagen I-coated vessels serve as a template for new bone. To test this hypothesis directly, we studied the mineralization of these vessels. To achieve this, *VECad-Cre, tdTomato* pregnant females were injected with calcein daily starting from E13.5. Calcein labeling enabled us to monitor the process of mineral deposition and to distinguish between old and newly deposited mineral by the intensity of fluorescent signal. As seen in Fig. 8 and Fig. S3, we observed areas of low calcein signal, suggesting newly forming bone. Interestingly, *tdTomato* expression was also observed in the same domains. At higher magnification, calcein was clearly detected around blood vessels. These results suggest that blood vessels in developing bones serve as a template for mineral deposition.

### Vascular patterning plays a role in bone morphogenesis

**Fig. 9.**
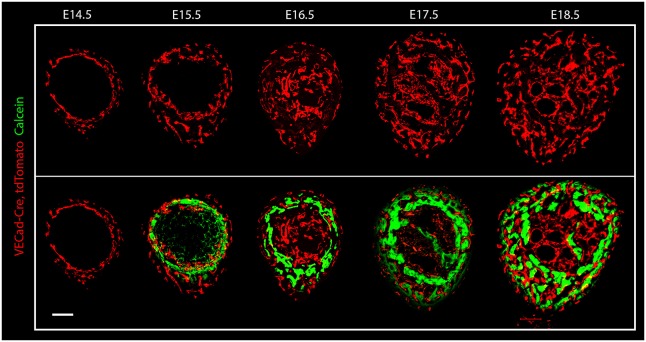
**Vascular and mineral distribution during bone development.** Cryostat cross-sections of humeri from *VECad-Cre, tdTomato* mouse at different embryonic stages (E14.5-E18.5). Blood vessels are visualized in red; calcein (green) was injected to visualize mineral deposition. Scale bar: 100 µm.

Our finding that blood vessels in the bone undergo mineralization led us to hypothesize that the vasculature plays an active role in bone morphogenesis. This notion implies that vascular patterning and bone growth are highly correlated: the former precedes the latter and determines the sites of mineralization. In order to test this hypothesis, the temporal and spatial distribution of both tissues was analyzed at different embryonic stages (Fig. 9). Mineral deposition was detected by calcein injection and the vasculature was visualized using *VECad-Cre, tdTomato* mice.

Comparison of vascular and mineral patterning revealed a correlation between the spatial distributions of the two tissues; however, intriguingly, the vascular patterning was always temporally one step ahead of mineral deposition. As seen in Fig. 8, although at E15.5 blood vessels were more broadly distributed than the mineral deposition, the vascular pattern at that stage was similar to the mineral deposition pattern observed at E16.5. The same time-delayed correlation persisted until E18.5, when embryonic circumferential bone growth has completed. These observations show that vascular patterning constantly precedes and predicts the sites of mineralization during development.

**Fig. 10.**
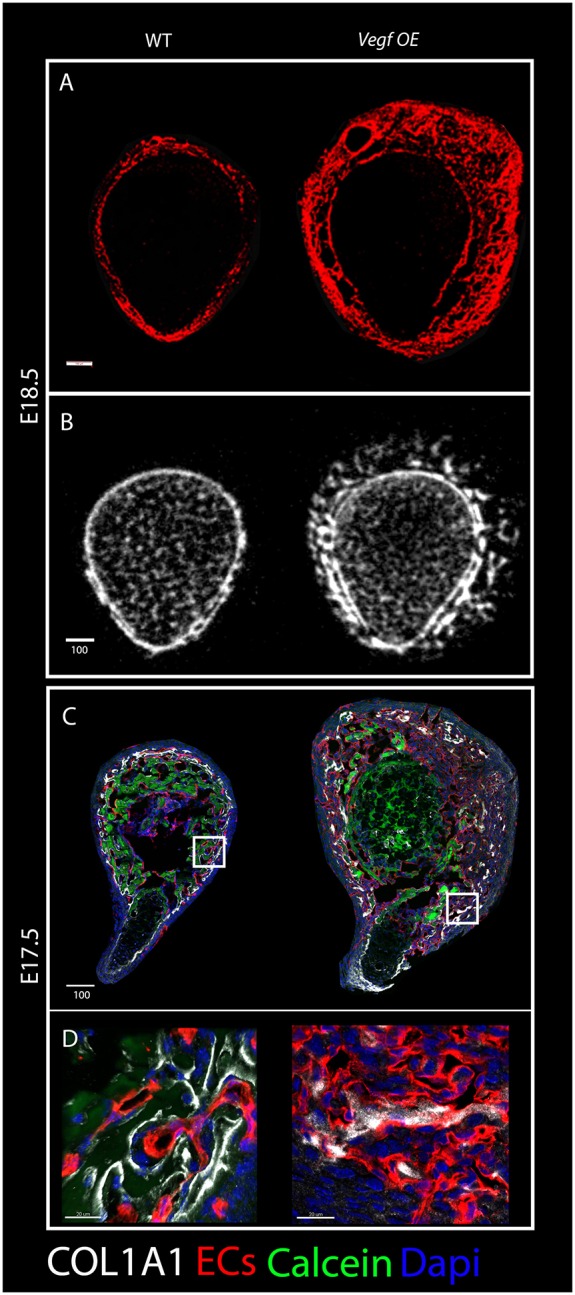
**Bone malformations upon *Vegf* overexpression.** (A) Cryostat cross-sections of humeri from wild-type and *Vegf*-OE mice at E18.5 immunostained for blood vessels (red) show expansion of the vascular patterning domain in the mutant. (B) Cross-sectional views of three-dimensional reconstructions from micro-CT scans of humeri from E18.5 wild-type (left) and *Vegf*-OE (right) mice illustrate the abnormal arrangement of the primordial cortex in the mutant. (C) Cross-sections of E17.5 humeri immunostained for blood vessels (red) and COL1A1 (white) show discontinuous collagen type I distribution in the mutant. (D) Magnifications of the boxed areas demonstrate collagen I association with ECs in both wild type and mutant. Scale bars: 100 µm in A-C; 20 µm in D.

To further demonstrate the effect of the vasculature on bone morphology, we interfered with vascular patterning and examined bone formation. To achieve this, we used a gain-of-function approach and overexpressed *Vegf* in osteoblasts using a triple transgenic system, in which the expression of reverse tetracycline transactivator (*rtTA*) and tetracycline-responsive element (*tetO-Vegf_165_*) was induced by *Col1a-Cre* mice carrying a promoter specific to osteoblasts (Belteki et al., 2005; Dacquin et al., 2002; Dor et al., 2002). *Vegf* overexpression (OE) was induced by doxycycline administration from E13.5 to E18.5. As expected and as previously shown (Maes et al., 2010a), *Vegf* OE led to altered vascular patterning, over-sprouting and high vessel density at the circumference of the bone, resulting in expansion of the vascularized area (Fig. 10A). Micro-CT analysis showed that bone formation followed suit, as the bone circumference expanded correspondingly (Fig. 10B). Analysis of *Col1a1* expression in *Vegf* OE bones revealed changes in its distribution, as small isolated islands of *Col1a1* were seen along the vasculature (Fig. 10C,D). As expected, collagen I was associated with ECs, suggesting that the changes in vascular patterning affected collagen distribution and thereby mineral deposition and bone shaping. These findings strengthen our conclusion that vascular patterning regulates bone morphogenesis by guiding collagenous template formation and mineral deposition.

## DISCUSSION

Previous studies have firmly established the importance of bone vascularization during skeletogenesis. Here, we identify a new role for blood vessels in regulating bone ossification by serving as the template for mineral deposition, implying that vascular patterning contributes to bone morphogenesis. The morphology of endochondral bones is defined by both longitudinal and circumferential growth. Previously, we described the appositional growth of developing bones through the formation of strut-ring layers (Sharir et al., 2011). Here, we show that blood vessels may contribute to the circumferential growth. Our finding that the pattern of blood vessel distribution along the cortex predicts the pattern of subsequent mineral deposition implicates the vasculature in bone morphogenesis. Moreover, changes in vascular patterning induced by *Vegf* overexpression resulted in correlative changes in ossification, further supporting this hypothesis. Although different mechanisms may be involved, this finding is in agreement with previous work showing that the vasculature plays active roles in morphogenesis of other tissue, such as the pancreas and lungs (Cleaver and Dor, 2012; Jakkula et al., 2000; Lazarus et al., 2011; Magenheim et al., 2011).

As we show, the ability of blood vessel to affect bone morphology relies on the fact that ECs in the developing bone are coated with collagen type I and, thus, serve as part of the osteoid template. This raises the question of what happens to the ECs that undergo collagen I coating and mineralization? Our study does not provide a direct answer to this. Yet, reduction in tdTomato signal could indicate that these cells undergo cell death. Another interesting issue relates to the observation that only part of the vasculature serves as a template for bone deposition. There are several possible mechanisms that may determine which vessels will undergo mineralization and which will retain their integrity and function. In the extracellular matrix, collagen I interacts with collagen-binding proteoglycans such as decorin, fibromodulin and lumican, whereas on the cell surface it can interact with integrins such as α_2_β_1_ (Calderwood et al., 1997; Danielson et al., 1997; Li et al., 2003; Nykvist et al., 2000; Velling et al., 2002). This integrin, which is the main receptor for collagen I, was shown to be expressed on ECs and it plays a role in angiogenesis (Pozzi et al., 2000; Tulla et al., 2001; Zhang et al., 2008). It will be interesting to study the involvement of this integrin in the mechanism that mediates the interaction between blood vessels and collagen I.

Another possible mechanism for mediating collagen-EC interaction is the ability of collagen I to interact with the platelet membrane protein glycoprotein (GP) VI. During thrombus formation upon vascular injury, GPVI plays a major role in the initiation of platelet aggregation through binding to collagen I (Jung et al., 2009; Moroi and Jung, 2004). It is therefore tempting to speculate that the bone capillaries, which lack BM, might be unstable leading to vessel leakiness and initiation of a coagulation-like process.

Our finding that bone vessels lack BM raises the question of what mechanism regulates BM formation or degradation? To date, the regulation of BM formation is poorly understood. Matrix metalloproteinases (MMPs) play a major part in the degradation of extracellular macromolecules such as collagen IV. Moreover, MMPs such MMP9 and MMP13 have been shown to play a role in endochondral bone formation. It is therefore reasonable to assume that these enzymes may be involved in the degradation of the BM in bone vasculature.

Interestingly, it has previously been demonstrated in bones of adult rat that, at the chondro-osseous junction, blood vessels have no BM either (Hunter and Arsenault, 1990). This suggests that a similar mechanism to the one we identify in the embryo may act in postnatal bone growth. A recent study showed that there are two subtypes of blood vessels in postnatal mouse bones. One of which, termed H type, is located at the chondro-osseous junction. H type ECs were shown to be associated with osteoprogenitor cells, coupling angiogenesis to osteogenesis (Kusumbe et al., 2014; Ramasamy et al., 2014). It would be interesting to examine whether the H type vessels lack BM and, if so, what role this absence plays in the ability of the vasculature to regulate bone growth.

**Fig. 11.**
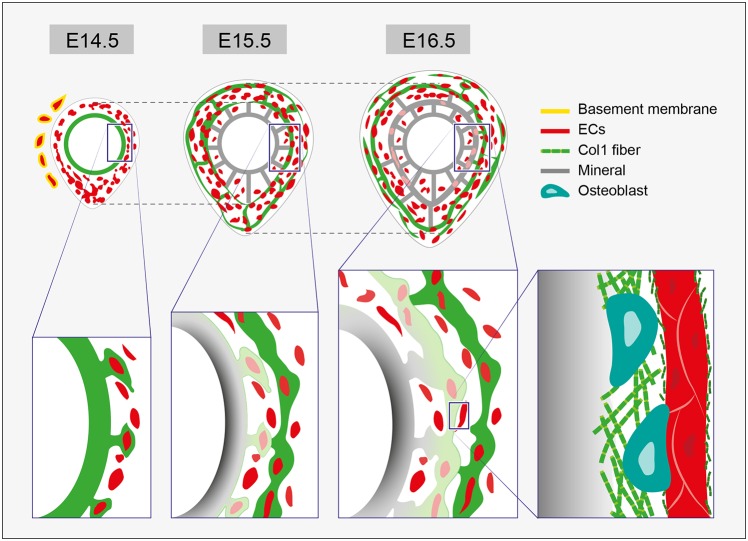
**Bone vasculature serves as a template for mineral deposition.** The involvement of vascular patterning in bone morphogenesis. Vascular patterning in the bone precedes and predicts mineral deposition sites. At E14.5, blood vessels are asymmetrically distributed. At E15.5, mineral distribution follows the vascular pattern of the previous day. Whereas blood vessels outside the bone are covered with basement membrane (BM), inside the bone the vasculature lacks BM. Enlargements of the boxed areas are shown below. At E14.5, collagen I is seen on the bone collar and on adjacent blood vessels. At E15.5, mineral is deposited onto the collagen I-covered vessels, which exhibit a reduction in EC marker. On the right is an enlargement of the boxed area showing older mineral undergoing thickening. Osteoblasts secrete collagen I, which is deposited on the mineral from one side and, in small fragments, on the blood vessel on the other side.

Our study suggests a new model for the involvement of the vasculature in bone morphogenesis (Fig. 11). Following the invasion into the cartilage, blood vessels are asymmetrically distributed in a stereotypic pattern. The vessels are then coated with collagen I that is secreted by adjacent osteoblasts, thereby contributing to osteoid formation. Thus, vascular patterning constantly precedes and predicts the sites of the next step of mineralization. As development proceeds, the vasculature expands circumferentially, resulting in the formation of strut-ring layers of new mineral. In the same manner, older mineral thickens, as osteoblasts continue to deposit mineral onto adjacent collagen I-covered vessels.

Here, we show for the first time that the endothelium serves as a template on which bone-forming cells build new bone tissue, implying that vascular patterning directs bone formation. These findings broaden our understanding of the contribution of blood vessels to bone development. In addition, this new concept may have clinical implications in conditions characterized by reduced bone mass, such as osteoporosis and aging.

## MATERIALS AND METHODS

### Mice

For genetic labeling of blood vessels, *Rosa26-tdTomato* mice (B6;129S6-*Gt(ROSA)26Sortm9(CAG-tdTomato)Hze*/J) (Madisen et al., 2010) were mated with mice expressing Cre recombinase under the control of the endothelium-specific promoter VE-Cadherin (*VECad-Cre*; Jackson Laboratories).

To switch *Vegf* overexpression on and off in the developing skeleton, we used a triple transgenic mouse line. We crossed heterozygous *Col1a-Cre* mice carrying a promoter specific to osteoblasts (Dacquin et al., 2002) with mice heterozygous for reverse tetracycline transactivator (rtTA) (Belteki et al., 2005) and tetracycline-responsive element (tetO)-driven transgene (*tetO*-*Vegf*) (Dor et al., 2002). Overexpression was induced by adding 200 μg/ml doxycycline hydrochloride (Sigma, D9891) in 3% sucrose to the drinking water of pregnant females from embryonic day (E) 13.5 until E15.5-E18.5. As a control, wild-type littermates were used. All animal experiments were pre-approved and supervised by the Institutional Animal Care and Use Committee (IACUC) of the Weizmann Institute.

In all timed pregnancies, plug date was defined as E0.5. For harvesting of embryos, timed-pregnant female mice were sacrificed by cervical dislocation. Tail genomic DNA was used for genotyping by PCR.

### Evaluation of bone deposition

Bone deposition was evaluated by daily intraperitoneal injections of calcein (Sigma, C0875; 2.5 mg/kg body weight) into pregnant females at E13.5-E18.5. Harvested limbs were fixed overnight in 4% paraformaldehyde (PFA) in phosphate-buffered saline (PBS), washed overnight in 30% sucrose/PBS at 4°C, embedded in OCT compound (Tissue-Tek) and sectioned by cryostat at a thickness of 10 µm. Fluorescence was visualized by confocal microscopy (LSM 780, Zeiss).

### Micro-CT analysis

Three-dimensional high-resolution images were obtained from the skeletons of *Col1a1-Cre*-*rtTA-tetO-Vegf* and control embryos using an eXplore Locus SP micro-CT scanner (GE Healthcare). Samples were scanned *ex vivo* in PBS solution at 45 kVp and 120 μA. For all scans, 900 projections over 360°, with four frames averaged for each projection at an exposure time of 2850 ms per frame, resulted in an isotropic voxel size of 6.731 μm. Voxel intensity was represented by data type int16. Calibration hydroxyapatite phantoms (GE Medical) were used to facilitate conversion of the linear attenuation of a given voxel to mgHA/cm³. Image reconstruction, thresholding (one threshold was chosen for all specimens) and measurements of morphological traits were conducted with MicroView software. For sample preparation, harvested bones were fixed overnight in 4% PFA/PBS at 4°C. After fixation, tissues were dehydrated to ethanol (25%, 50%, 75%, 100%) and stored at −20°C until scanning.

### Electron microscopy

Humeri of E16.5 mouse embryos were fixed in a freshly prepared 4% PFA and 2% glutaraldehyde in 0.1 M cacodylate buffer containing 5 mM CaCl_2_ (pH 7.4) for 4 h at room temperature. Samples were kept at 4°C overnight in the same fixative. The tissue was then dissected and fixed again in the same fixative at 4°C. After washing, tissue was post-fixed in 1% osmium tetroxide, 0.5% potassium dichromate and 0.5% potassium hexacyanoferrate in cacodylate buffer for 1 h, and incubated in 2% (w/v) uranyl acetate for 1 h. The tissues were embedded in EMbed 812 (EMS). Thin sections were cut using a diamond knife (Diatome), stained with 2% uranyl acetate and Reynold's lead citrate, and examined with transmission electron microscope FEI Spirit CM12 at an accelerating voltage of 120 kV and recorded with an Eagle CCD camera.

### Cryosectioning and immunogold labeling for electron microscopy

For immunogold electron microscopy, tissues were processed using a standard procedure (Tokuyasu, 1980). Fixation was performed with freshly prepared solution of 4% PFA, 0.1% glutaraldehyde in 0.1 M cacodylate buffer containing 5 mM CaCl_2_ for 4 h at 4°C on the shaker. Fixed tissue was infiltrated in 10% gelatin at 37°C for 30 min, then excess of gelatin was removed at 37°C, followed by post-fixation at 4°C for 24 h. Post-fixed tissue was dissected to small pieces, cryoprotected by overnight infiltration with 2.3 M sucrose in cacodylate buffer. Samples were then frozen by liquid nitrogen and ultrathin sections (75 nm) were cut with a diamond knife at −110°C on Leica EM FC6 cryo-ultramicrotome and transferred to formvar-coated 200-mesh nickel grids. Sections were treated with blocking solution (0.5% bovine serum albumin and 0.1% glycine in PBS) for 15 min to block non-specific binding, followed by 2 h incubation with the primary antibody anti-collagen I (1:5; Abcam, ab21286). After extensive washing in PBS/0.1% glycine, the grids were incubated with the secondary antibody 10 nm colloidal gold-conjugated goat anti-rabbit IgG (1:20; EMS, 25109) for at least 30 min at room temperature. The grids were then washed in PBS-glycine, stained with neutral uranyl acetate oxalate for 5 min, briefly washed and stained with 2% uranyl acetate in H_2_O for 10 min, and then embedded in 2% methyl cellulose/uranyl acetate as described previously (Tokuyasu, 1980).

### Serial block-face scanning electron microscopy (SBF-SEM)

Samples of E16.5 bone were prepared for SBF-SEM as described previously (Starborg et al., 2013) using a Gatan 3View microtome within an FEI Quanta 250 scanning microscope. The 3View microtome removed 1000×100 nm sections from sample blocks. The *x* and *y* axes of each image were equivalent to 4096×4096 11 nm pixels. Object segmentation was performed manually using IMOD (Kremer et al., 1996).

### Immunofluorescence

For cryosection immunofluorescence, freshly dissected limbs were fixed overnight in 4% PFA, transferred to 30% sucrose overnight, then embedded in OCT and sectioned by cryostat at 10 µm. Cryosections were dried and post-fixed for 30 min in 4% PFA and permeabilized with 0.2% Triton/PBS. To block non-specific binding of immunoglobulin, sections were incubated with 7% goat serum in PBS. Cryosections were then incubated overnight at 4°C with primary antibodies: rat anti-mouse CD31 (BD PharMingen, PMG550274; 1:50), rat anti-endomucin (Santa Cruz, sc65495; 1:100), rabbit anti-mouse collagen type I antibody (EMD Millipore, AB765P; 1:100), rabbit anti-collagen I (Abcam, ab21286; 1:100), rabbit anti-NG2 (Millipore, AB5320; 1:50), rabbit α-smooth muscle actin (αSMC) (Novus, NB 600-531; 1:200), rabbit anti-mouse OSX (A-13)-R antibody (Santa Cruz, sc-22536-R; 1:200), mouse anti-laminin [Developmental Studies Hybridoma Bank (DSHB), 2E8; 1:10], mouse anti-fibronectin 1H9 Fibronectin Hep2/1H9B2 (DSHB, 1H9; 1:10) and rabbit anti-collagen antibody, type IV (EMD Millipore, AB756P; 1:100). Sections were washed in PBS and incubated with secondary fluorescent antibody Alexa Fluor 488-AffiniPure donkey anti-rabbit IgG (Jackson Laboratories, 711-545-152; 1:100). Samples were then washed and mounted on glass slides and examined with LSM 780 laser-scanning confocal microscope (Carl Zeiss).

### *In situ* hybridization

Double fluorescence *in situ* hybridization on cryosections were performed using fluorescein- and DIG-labeled probes. After hybridization, slides were washed, quenched and blocked. Probes were detected by incubation with anti-fluorescein-POD and anti-DIG-POD (Roche; 1:200), followed by Cy3- and Cy2-tyramide-labeled fluorescent dyes (according to the instructions of the TSA Plus Fluorescent Systems Kit, PerkinElmer). *tdTomato* antisense probe was generated using the following primers: forward, tcccacaacgaggactacaccat; reverse, cgcgcatcttcaccttgtagatca. The *Col1a1* probe was a 183 bp fragment of the carboxyl propeptide domain. The extended protocol is described elsewhere (Shwartz and Zelzer, 2014).

### Super-resolution stochastic optical reconstruction microscopy (STORM)

For sample preparation, E15.5 mouse embryos were dissected and forelimbs were fixated with PFA 4% overnight at 4°C. Then, forelimbs were dehydrated with 30% sucrose overnight at 4°C and embedded in OCT compound. Cryostat cross-sections (10 µm) were mounted on 1.5H (0.170 mm) round coverslips coated with 0.1% gelatin. Samples were incubated at 37°C overnight. Following the immunostaining protocol described above, samples were immunostained for collagen I (1:50) and endomucin (1:100) using the secondary antibodies Cy3b anti-rabbit (1:50; antibody preparation is described below) and goat anti-rat Alexa 647 (1:800; Thermo Fisher, A-21247). Samples were washed with PBS and analyzed.

STORM imaging was performed using Vutara SR-200 microscope. Coverslips were placed in a Petri dish in imaging buffer containing 7 μM glucose oxidase (Sigma), 20 mM cysteamine (Sigma), 150 mM β-mercaptoethanol (Sigma), 50 mM Tris, 10 mM NaCl, 56 nM catalase (Sigma), 10% glucose (pH 8.0). Alexa 647 was excited with 647 nm laser power at a range of 4-9 kW/cm^2^ and Cy3b was excited with 561 nm laser power of about 6 kW/cm^2^. To maintain optimal single-molecule density, 405 nm activation laser power was ramped slowly. *Z*-stack was performed by acquiring 700 frames at 50 Hz for each *z* position at 0.1 µm steps. Single-molecule fitting was performed using Vutara software. The extended protocol is described elsewhere (Rust et al., 2006).

For Cy3B conjugation to a secondary IgG antibody, Cy3B NHS ester (0.02 mg; GE Healthcare, PA63101) was dissolved in 10 µl DMSO. AffiniFure donkey anti-rabbit IgG (H+L) (≈1.25 mg/ml; Jackson ImmunoResearch, #711-005-152) was mixed with 1.5 μl of Cy3B NHS ester solution and with 6 μl of 1 M NaHCO_3_ (pH 8.3) for 30 min at room temperature in the dark. Illustra NAP-5 Columns (GE Healthcare, 17-0853-02) were equilibrated by running three column volumes (∼10 ml) of PBS. PBS (140 μl, pH 7.2) was added to the antibody reaction and mixed gently, yielding a final volume of 200 μl. The antibody reaction was added to the center of the NAP-5 column. After all the antibody mix fully entered the column, the column was washed with 550 μl PBS. Once the wash had stopped dripping from the column, 300 μl PBS was added and the eluent was collected.
